# Object size can influence perceived weight independent of visual estimates of the volume of material

**DOI:** 10.1038/srep17719

**Published:** 2015-12-02

**Authors:** Myrthe A. Plaisier, Jeroen B.J. Smeets

**Affiliations:** 1 Department of Human Movement Sciences, Research Institute MOVE, VU University, Amsterdam, The Netherlands

## Abstract

The size-weight illusion is the phenomenon that the smaller of two equally heavy objects is perceived to be heavier than the larger object when lifted. One explanation for this illusion is that heaviness perception is influenced by our expectations, and larger objects are expected to be heavier than smaller ones because they contain more material. If this would be the entire explanation, the illusion should disappear if we make objects larger while keeping the volume of visible material the same (i.e. objects with visible holes). Here we tested this prediction. Our results show that perceived heaviness decreased with object size regardless of whether objects visibly contained the same volume of material or not. This indicates that object size can influence perceived heaviness, even when it can be seen that differently sized objects contain the same volume of material.

It has been suggested that when the expectation about the object’s mass does not match the physical value, an illusory heaviness percept can occur^1^. Such a mismatching expectation can be based on the object properties such as surface material or size; the latter case results in the size-weight illusion^2^ (recently reviewed by Buckingham^3^). The expectation that a larger object will feel heavy is presumably caused by the fact that larger objects generally contain more material and consequently have a larger mass than smaller objects. When, in contrast, the two objects have the same mass, an illusory heaviness difference occurs.

Recent support for the idea that the size-weight illusion is caused by a mismatch between expected and actual mass is given by a study in which participants were only shown an object prior to lifting, but not during. In that case they still experienced a size-weight illusion^4^. Visual information during lifting is thus not necessary to elicit the size-weight illusion, supporting the idea that the size-weight illusion is based on an expectation about the heaviness (or actually the mass^5^,^6^) of an object before lifting. That this expectation is likely based on an inferred relationship between size and mass has been demonstrated by Flanagan and colleagues using a learning paradigm^7^. In that study participants were asked to repeatedly lift objects from a set in which there was an inverse correlation between the size and mass of the objects (i.e., the smaller objects had a larger mass than the larger objects). During several days to weeks of training the size-weight illusion reduced and ultimately even reversed. So it is reasonable to assume that the size-weight illusion finds its origin in the correlation between the size and the mass of objects.

For many sets of objects made out of the same material the mass of the objects correlates with the size of the objects, i.e. the distance between the outer edges. Research into the size-weight illusion has been mainly conducted with sets of objects that appear to be homogeneous and solid, such that the size of the objects is directly related to the volume of material. However, if a set consists of objects that have holes in it, a larger object can have a smaller volume of material and thus be less heavy than a smaller object. Here we investigated whether a weight illusion can also occur when it is clearly visible that the size of an object does not correlate with the volume of material (and therefore its mass) contained in the object (Fig. 1). If so, this would indicate that a direct relationship between size and expected weight of objects, bypassing the estimation of the volume of material, plays role in heaviness perception.

## Results

### Experiment 1

**Figure 1:**
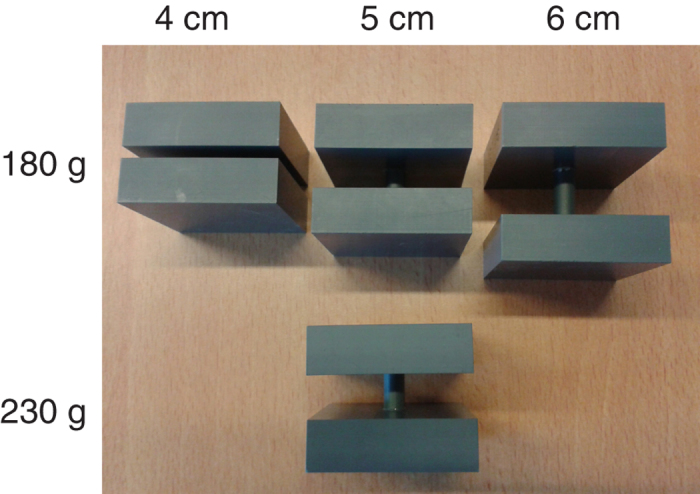
A set of objects that visually contain the same volume of material, but differ in size (spacer-objects). The objects were constructed out of two 6 × 6 × 1.8 cm slabs of PVC. Object size was varied by varying the length of the spacer between the two slabs. In Experiments 1 and 2 we used three 180 g objects varying in size (4 cm, 5 cm and 6 cm) and one 230 g object of 5 cm, in Experiment 3, two more 180 g objects (4.5 cm and 5.5 cm, not shown) were added to the set.

In the first experiment we tested whether the size-weight illusion is based on information about the volume of material in an object. Therefore, we determined the perceived heaviness of three objects that differed in size, but contained (visually and actually) the same volume of material: all objects consisted of two equally sized slabs of PVC separated by a spacer (Fig. 1). We will refer to these as spacer-objects. To vary the information about the volume of material, two conditions were performed: ‘haptic size only’ and ‘visual and haptic size’. The two conditions were presented interleaved. In both conditions participants grasped the object between thumb and index finger along the object axis that varied in size. They lifted the object and placed it back on the table. After each lift, participants gave a heaviness rating using a method of free magnitude estimation^8^. Participants wore a pair of shutter glasses that opened shortly before the beginning of a trial in the ‘visual and haptic size’ condition and remained closed in the ‘haptic size only’ condition. This means that in the ‘haptic size only’ condition, there was haptic size information available from the grip aperture used for lifting the objects, but participants had no direct cues indicating that the volume of material did not increase with the size of the object. In the ‘visual and haptic size’ condition participants had in addition to the haptic information visual information about size as well as about the volume of material, i.e. they could see the gap in between the two slabs of PVC. If the incorrectly anticipated mass causing the size-weight illusion is based on an estimate of the volume of material in an object, we expect that there will be no or a very weak illusion in the ‘visual and haptic size’ condition and an illusion of full magnitude in the ‘haptic size only’ condition.

**Figure 2:**
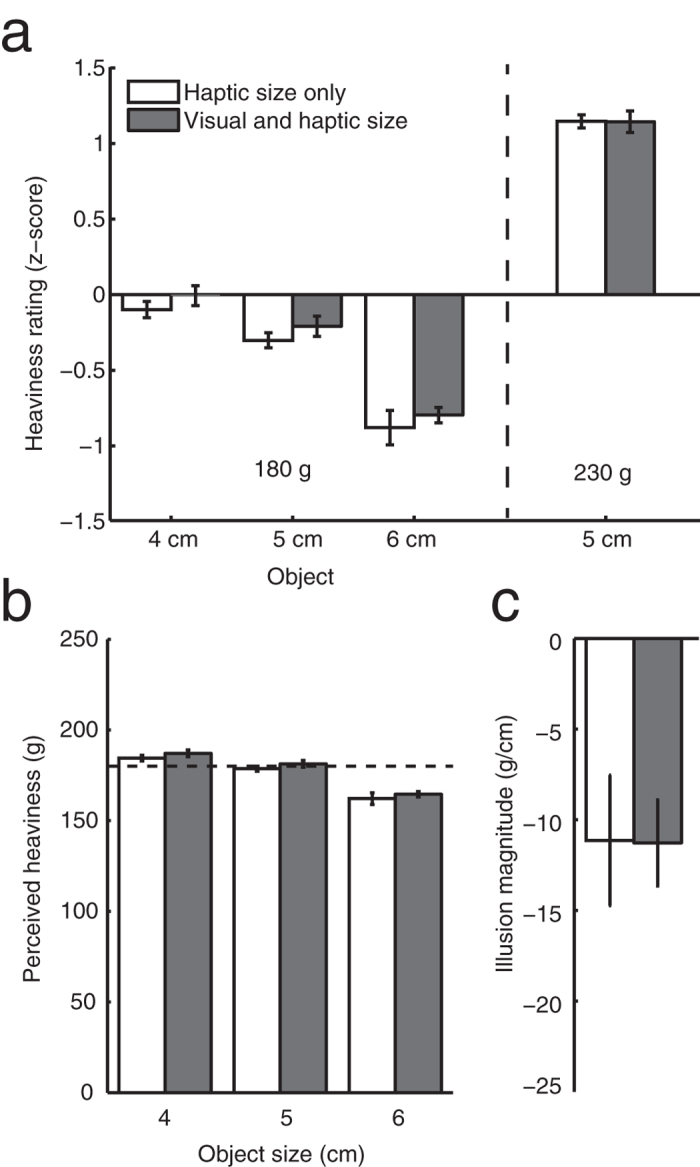
Results of Experiment 1, averaged over participants for the different object sizes in both conditions. Error bars indicate the between subjects standard error. (**a**) The z-scores of the heaviness ratings. (**b**) The perceived heaviness (heaviness ratings converted into units of grams). The dashed line indicates the mass of the objects. (**c**) The magnitude of the illusion (the slope of a linear fit to the perceived heaviness as a function of objects size for the 180 g objects). The fit was performed on the individual participants’ data. Error bars indicate the standard error.

The heaviness ratings of the subjects were given in arbitrary units. After converting them to z-scores (Fig. 2a) we determined the perceived heaviness in units of grams (Fig. 2b). Subsequent linear regression to the perceived heaviness as a function of object size yielded an illusion magnitude measure (Fig. 2c): the perceived increase of heaviness in grams per centimetre increase in size. Note that this measure will be negative as the illusion leads to a decrease in perceived heaviness with increasing object size (see Method section for details on data analysis). Repeated measures ANOVA on the perceived heaviness showed that there was indeed an effect of object size (F (2, 18) = 35, p < 0.0001), and no effect of trial type (F (1,9) = 1.7, p = 0.23), nor an interaction effect (F (2, 18) = 0.012, p = 0.99). The magnitude of the illusion was approximately −12 g/cm in both conditions.

One could argue that the volume of visible material of the larger objects was indeed larger than that of the small objects due to the material of the spacers. Could this be the basis of the measured illusion? Due to the spacer the increase of visible material between the smallest and largest objects was 0.44%, whereas if the object had been solid (as subjects will have assumed in the haptic size only condition) this increase would have been 50%. If the illusory heaviness difference would be based on an estimated volume of material, we would expect that the illusion magnitude in the condition with vision would be only about 0.88% of the magnitude of the illusion in the haptic size only condition. A paired t-test showed that was not the case: the illusion magnitude in the visual and haptic size condition was larger than 0.88% of the illusion magnitude in the haptic size only condition (t (9) = 5.6, p < 0.0001). This shows that the illusory heaviness difference was not based on a visual estimate of the visible volume of material in our experiment.

We find a clear illusory heaviness reduction consistent with the size-weight illusion for objects that have the same visual volume of material but differ in size. The illusion magnitude in the visual and haptic size condition was much larger than expected based on a visual estimate of the volume of material. This suggests that an estimate of the volume of material was not taken into account in this case. After the first couple of trials, however, participants could have realised that the same set of objects that was used in the visual and haptic size condition might also be used in the haptic size only trials, and thus that haptic size is not a valid cue for the volume of material. If so, the illusion in the haptic size only trials might therefore have been reduced by knowledge about the volume of material in a similar way as the visual and haptic size condition. This may be the reason why we did not find a difference between the conditions (although not very likely, given the very strong illusion). This explanation was investigated in Experiment 2.

### Experiment 2

In Experiment 2 we tested whether we can explain the results of Experiment 1 by assuming that participants experienced a considerably reduced weight illusion in both conditions of Experiment 1 due to visual information about the objects obtained from previous trials. If so, the magnitude of the illusory weight difference should be considerably larger if the subjects never see the objects. To this end we repeated the ‘haptic size only’ condition from Experiment 1 with a new group of participants. These participants had never seen the objects and were never shown the objects during the experiment.

If the magnitude of the illusion found in the ‘visual and haptic size’ condition of Experiment 1 was reduced due to the participants being aware of how the objects were constructed, the illusion should be larger in Experiment 2.

**Figure 3:**
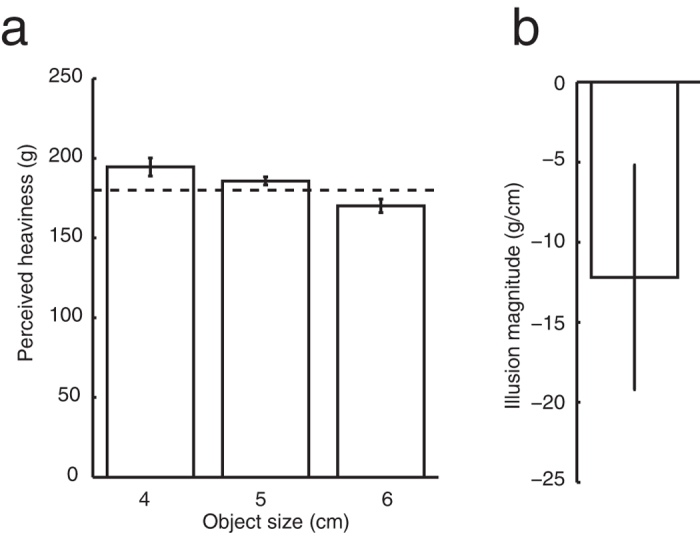
Results of Experiment 2. (a) Perceived heaviness (dashed line indicates the mass of the objects). (b) The magnitude of the illusion. Error bars indicate the standard error.

Upon debriefing all participants reported that they had assumed the objects were solid throughout the experiment. As expected, the perceived heaviness decreased with object size (Fig. 3a, F (2,18) = 14, p < 0.001). Most importantly, the magnitude of the illusion (−12 g/cm, Fig. 3b) is comparable to the value that was found in Experiment 1. If the illusion found in the haptic size only condition of Experiment 1 were a reduced value due to visual information from interleaved trials, we would expect the illusion to be 113 times larger here in Experiment 2. Clearly this was not the case, and for completeness this was also confirmed with an unpaired t-test (t (18) = 4.1, p < 0.0001).

The comparison of Figs 2c and 3b shows that the magnitude of the illusion in the haptic size only condition of Experiment 1 was not considerably reduced due to the interleaving trials with vision. Instead the illusion appears to have been of (almost) full magnitude in both conditions of Experiment 1.

We can conclude that subjects’ judgements were influenced by the distance between the outer edges rather then by a visual measure of the volume of material. It might be that subjects relied on haptic information about object size only. This argument is in line with the suggestion that haptic size information as perceived by handling an object is necessary for a full magnitude of the illusion to occur, because when objects are lifted with a tool such as a string, the illusion is reduced compared to using a direct grip^9^,^10^. In Experiment 3 we investigated whether the illusory weight differences found in Experiments 1 and 2 were due to haptic size information dominating all visual information.

### Experiment 3

**Figure 4:**
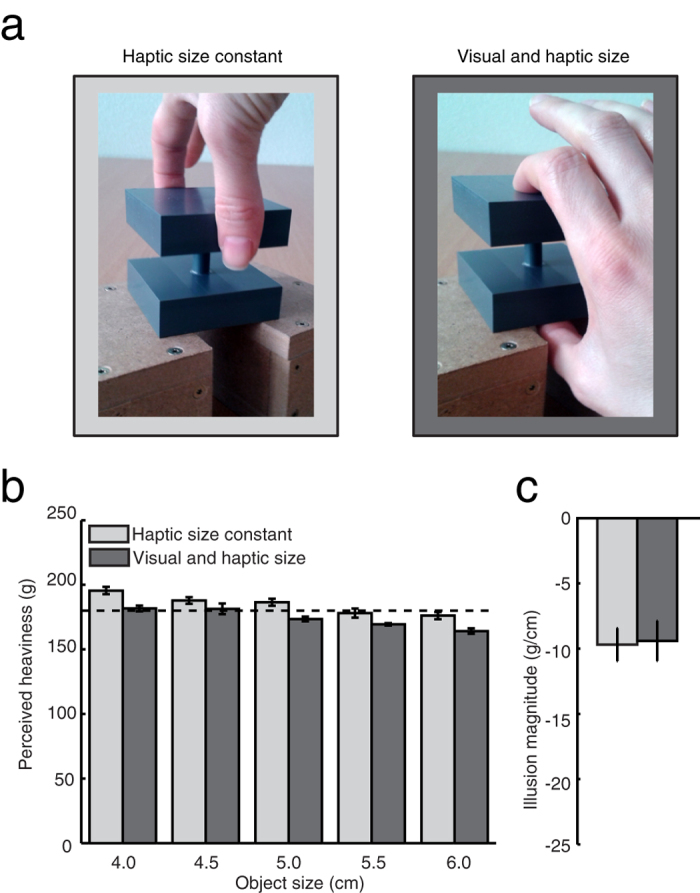
Experiment 3. (**a**) The two conditions: objects were grasped either along the vertical axis that co-varied in length with object size, or a horizontal axis that was the same length for all object sizes. (**b**) Perceived heaviness (the dashed line indicates the mass of the objects). (**c**) The magnitude of the illusion. Error bars indicate the standard error.

The size of our objects varied only along one axis, the other two axes were kept constant (6 cm). In this experiment we asked participants to lift the objects along one of the constant axes (‘haptic size constant’ condition), or along the variable axis (‘visual and haptic size condition). This means that participants used one hand to interact directly with the objects in both conditions (Fig. 4a). Therefore, participants in this experiment always had direct haptic size information about the object, but in one condition this haptic size information was the same for all objects while in the other it varied with object size.

The perceived heaviness decreased with object size in both conditions (Fig. 4b), indicating that the size-weight illusion is not only based on haptic information about object size. In addition, we found that in the haptic size constant condition all objects were rated to be heavier than in the visual and haptic size condition. Repeated measures ANOVA showed indeed an effect of object size (F (4, 36) = 19, p < 0.0001) and condition (F (1, 9) = 9.2, p = 0.014), and no interaction effect (F (4, 36) = 1.1, p = 0.36). The magnitude of the illusion was on average −10 g/cm in both conditions (Fig. 4c).

We did not have any expectations for a main effect of condition. The two conditions differ in the way the object is lifted (digits on the side of the object versus digits above and below the object) and the grip configuration must have affected the perceived heaviness. Previous research has indeed shown that grip configuration can influence perceived heaviness^11^. Importantly, in both conditions of the current experiment a size-weight illusion occurred and thus also when only visual size information was available. This shows that the illusory weight difference was not, or at least not completely, driven by haptic size information from differences in hand opening. The illusory weight difference between the differently sized objects can therefore not have been solely due to haptic information about size dominating all visual size information.

So far the results of each of the experiments indicated that visual information regarding the volume of material contained in an object does not greatly affect the magnitude of the size-weight illusion. In the next experiment we directly compared the perceived heaviness of a solid looking hollow object to a spacer-object.

### Experiment 4

**Figure 5:**
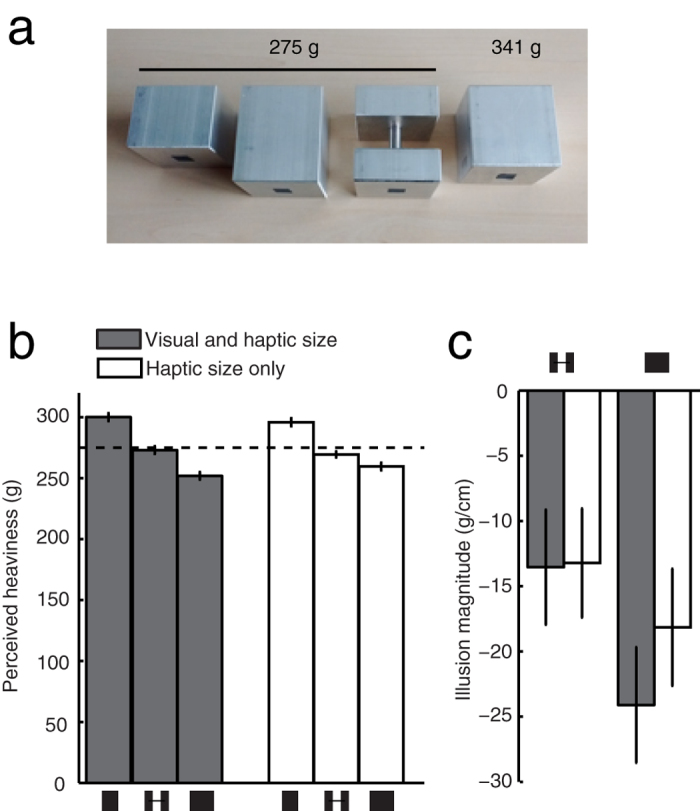
Experiment 4. (**a**) The four objects used in this experiment. The leftmost and rightmost object were solid, the middle two had holes (which were invisible in the right middle one). The left three objects were used to determine the size-weight illusion; the right object allowed us to express the heaviness ratings in grams. (**b**) The perceived heaviness in grams for both groups for the three objects of 275 g. (**c**) The illusion magnitude for both groups. Error bars represent the standard error across participants.

In this Experiment we used 4 objects made out of metal (see Methods for details). Two of these objects were made out of solid pieces of material and therefore varied in size and weight such as they would normally given the density of the material. The third object looked solid but was hollowed out and the forth object was a spacer-object which visually contained the same volume of material as the smallest object (Fig. 5a). Participants were asked to lift an object along the axis that differed in size between the objects and to rate the heaviness of the object using a method of free magnitude estimation. Two groups of participants were tested: one with full vision of the objects and one without vision of the objects.

The heaviness ratings in grams for both groups are shown in Fig. 5b. It can be seen that both groups of participants perceived the two larger objects as less heavy than the small object with the same mass. The magnitude of the illusory weight difference was larger for the solid looking hollow object than for the spacer object (Fig. 5c). To test whether this difference was significant, a mixed repeated measures ANOVA with vision as between subjects factor and object as within subject factor was performed on the illusion magnitude. This analysis showed indeed a main effect of object (F (1,38) = 23, p < 0.0001), without a significant main effect of vision (F (1,38) = 1.5, p = 0.2) or interaction between object and vision (F (1,38) = 3.4, p = 0.073).

Our results show that both the solid looking hollow object and the spacer-object were perceived to be less heavy than the smaller object of equal mass. This illusory reduction in heaviness was smaller for the spacer-object than for the solid looking hollow object, irrespective of whether the object was visible. The fact that also in the haptic-size only condition the size-weight illusion was reduced for the spacer-object indicates that the reduction of the size-weight illusion between these two objects was not predominantly driven by visual information about the amount of material. If we would interpret the lack of a significant interaction between object type and vision as indicating the absence of such an interaction, this would even suggest that there was no contribution of vision to the difference in illusion magnitude for the two objects. Thus, most likely the difference in the distribution of the mass between these two objects was the main cause of the smaller illusion magnitude for the spacer object.

## General Discussion

The results from Experiments 1 and 2 clearly show that a size-weight illusion occurs even for objects that visibly all contain approximately the same volume of material. Although containing the same volume of material is a direct cue indicating that all objects have the same mass, our participants apparently did not take this information into account to form their expectation of the object’s mass. The results of Experiment 3 show that the illusory weight differences were not caused by haptic size information dominating visual information. The illusion also occurred in the absence of haptic size differences indicating that visual size differences can also cause the illusory weight differences. In Experiment 4 we found a difference in illusion magnitude between a solid looking hollow object and a spacer-object of the same size (as defined by the outer edges of the object). Although both objects were perceived to be lighter than a smaller object of equal mass, this illusory weight difference was larger for the solid looking hollow object. This difference between the objects, however, persisted in the absence of vision indicating that it was not completely caused by visual information about the volume of material in the object.

The results of each of the four experiments in our study consistently indicate that size can influence perceived heaviness even if it can be seen that the volume of material in the object does not (or only very slightly) vary with size. The fact that the weight illusion did not appear to depend on whether participants could see the objects or not as in Experiments 1 and 2, suggests that visual information about the volume of material in the object did not cause the illusory weight difference. This was again confirmed in Experiment 4. Although the solid looking hollow object and the spacer-object did not yield the same illusory weight difference, this difference was found in the absence of visual information as well. This indicates that the perceived heaviness difference between the two objects was likely due to the distribution of the mass and not due to visual information about the volume of material in the object.

Our experiment 4 suggests that mass distribution plays a role in the size-weight illusion. It has been proposed that the size-weight illusion can be explained solely by differences in moments of inertia for differently sized objects^12^. Based on this moments of inertia reasoning, however, one would expect that the 6 cm object of the spacer-object set would be perceived to be heavier than the 5 cm object. Our data shows the opposite effect, as the 6 cm object is rated to be lighter than the 5 cm object. Therefore, a direct scaling of perceived weight based on the moment of inertia of objects cannot explain our finding that within the set of spacer-objects the perceived heaviness decreased with increasing object size.

In this study we have shown that size can influence perceived heaviness largely independent of the volume of material that is visible in an object. In these cases, the size of an object appears to be, treated as directly related to object mass regardless of the volume of material. This suggests that this inferred relationship between size and mass of an object can dominate, or possibly overwrite, certain other visual cues indicating that objects have in fact the same mass. A possible explanation for such dominance of size information would be that most objects we handle are solid. Because in that case size is directly correlated to the volume of material in the object it makes sense that we would have learned a relationship between size and mass irrespective of the volume of material.

It has been suggested that density information, plays a role in the size-weight illusion^14^ and it is suggested to be a result of the densities of the two differently sized objects being unexpectedly different^15^,^16^. Of course, in experiments usually one of the two objects is manipulated to achieve equal masses despite size variations. It has recently been shown that the density of objects tends to vary with the size of objects and that the brain infers this statistical relationship from the environment which leads to the size-weight illusion^17^. Our results, however, indicate that perceived heaviness also decreases with object size even when it can be seen that the object is mostly hollow in the centre, thus indicating that a direct inference between size and weight can also play a role in heaviness perception.

## Methods

### Participants

All participants were recruited among employees and students of the Department of Human Movement Sciences. They were randomly assigned to one of the experiments. Thirty participants were randomly assigned to either Experiment 1, 2 or 3 such that ten participants participated in each of these experiments. Another forty participants (20 per group) took part in Experiment 4. Seven of the participants in the no vision group had also participated in either Experiment 1 or 2. All participants were naive as to the purpose of the experiment. They had normal or corrected to normal vision and none of the participants reported any known somatosensory deficits. Informed consent was obtained from all participants prior to participation in the experiments. The experiment was part of a program that was approved by the ethical committee of the Faculty of Behavioural and Movement Sciences at VU University. The experiments were carried out in accordance with the approved guidelines.

### Stimuli

Objects for Experiments 1, 2 and 3 were constructed out of two slabs of PVC (6 × 6 × 1.8 cm). These slabs were the same size for all objects, and an object consisted of two of these slabs connected using thin PVC spacers (0.6 cm diameter) varying in length. By pushing the shorter spacer further into the holes in the PVC slabs, the visible length of the spacer varied while the actual length of the spacer varied much less. This ensured that the mass difference between the objects was less than 1%. Therefore, the set of objects had a uniform mass and varied in size along a single dimension (Fig. 1). By choosing equally shaped slabs of PVC for all objects, correctly estimating the volume of material between the different objects is facilitated, as using differently shaped objects can lead to biases in perceived volume and weight^13^.

In Experiments 1 and 2, the objects were presented in the orientation as shown in Fig. 1, and always grasped along the variable axis. In these experiments we used four objects from this set: one 4 cm object, two 5 cm objects and one 6 cm object. One of the two 5 cm objects was weighed down with lead such that its mass was 230 g (this was not visible to the participants). The other three objects all had a mass of 180 g. In Experiment 3 we additionally used a 4.5 cm and a 5.5 cm object of 180 g from the set. In this experiment, we enabled the objects to be grasped along a constant and along the variable axis while keeping their orientation the same by rotating the objects 90° with respect to the orientation used in Experiments 1 and 2 such that the variable axis was vertical (Fig. 4a). In Experiment 4 we used a set of objects made out of metal (Duralumin, 2.8 g/cm^3^). Two objects varied in size and weight consistent with the density of the material: small object (4 × 5 × 5 cm, 275 g) and a larger one (5 × 5 × 5 cm, 341). There were also two larger objects. One of these looked solid but was hollowed out in the centre (6 × 5 × 5 cm, 275 g) and the other one was a spacer-object consisting of two slabs of metal with a 2-cm spacer (0.7 cm diameter) in between (6 × 5 × 5 cm, 275 g). Participants always grasped the objects along the variable axis. Because the metal was quite smooth we attached two small patches of sand paper (1 × 1 cm) at the grasping locations to facilitate grasping.

A small visual control experiment was conducted to assess how well participants could visually judge the volume of material in the spacer-object. For the visual control experiment we used a set of comparison objects made out of solid pieces of Duralumin with a cross section of 5 × 5 cm and that varied in length from 2 to 6 cm in steps of 0.5 cm. Nine participants were recruited among the department staff for a very quick assessment of how well participants would be able to compare the volume of material in solid objects to a spacer-objects. The participants were shown a set of comparison objects and were asked to indicate which of these contained the same volume of material as the simultaneously shown 6-cm spacer-object. Participants were quite accurate at this task as the answers varied only between the 4 and the 4.5 cm comparison objects. The average was 4.3 ± 0.09 (SE) cm. This is a slight overestimation as the two slabs of material divided by the spacer were equivalent to the 4 cm solid object. Even if the spacer was taken into account this was an overestimation given that the spacer had a volume of only 0.8 cm^3^ so the solid object with equivalent volume of material would have had to be 4.04 cm long. This indicates that participants were relatively accurate at comparing the volume of material between a solid and spacer-object, but tended to overestimate the volume of the spacer-object by 7.5%.

### Experimental design

In all experiments each object was presented 10 times in each experimental condition. In Experiment 2 the conditions were performed in separate blocks while in the other experiments conditions were randomly interleaved. In experiment 4 we presented the objects in random order. In order to ensure that all conditions were distributed over the experiment (or block), the objects were presented in groups of trials in the other experiments. In every group of trials, each of the objects was presented once per condition. Participants were never aware that the objects were presented in trial groups, nor were they aware of the total number of objects in an experimental set.

Participants were seated at a table on which the objects were presented. In Experiment 1 they wore a pair of liquid crystal shutter glasses (Plato System; Translucent Technologies, Toronto, Ontario, Canada). The set of objects was always obscured from the participant’s view prior to the experiment and in between trials. This ensured that participants never saw more than one object at the same time and they were not aware of the total number of objects in the set. In Experiment 2 and the no vision condition of Experiment 4 the objects were presented behind a curtain such that they were obstructed from view. Participants were instructed to grasp the object between thumb and index finger and to smoothly lift the object to a height of approximately 20 cm and place it back on the table. On trials without vision the participants waited for a verbal signal from the experimenter indicating they could grasp and lift an object. The objects were always placed in the same location such that participants could easily grasp them in the absence of vision. Prior to each of the experiments participants performed a couple of practice lifts with an object that was not part of the experimental set to become familiar with the task.

### Analysis

The magnitude of the illusion was calculated by first converting for each participant all heaviness ratings in an experiment from arbitrary units into *z*-scores to be able to compare between participants. All statistical tests were performed on the z-scores. To obtain values that are easier to interpret, we subsequently converted the z-scores into perceived heaviness (in units of grams) based on the difference in z-score between objects that actually differed in mass. In Experiments 1–3 this was done based on the *z*-scores for each of the two 5 cm objects for which there was a physical mass difference. In case there was more than one condition (Experiments 1 and 3) the z-scores for the two 5 cm objects were averaged over the conditions. The average *z*-score for the 5 cm object of 180 g was set to represent 180 g perceived mass. The difference between the average *z*-scores of the two 5 cm objects was divided by the physical mass difference between the two objects (50 g). This ratio was determined on the population level and used to transform the *z*-score values for all objects in each of the conditions into perceived heaviness. In principle this is only an axis transformation of the z-scores. The magnitude of the size-weight illusion in these experiments was determined by a linear fit to the perceived heaviness as a function of object size, resulting is an illusion magnitude measure in units of g/cm.

In Experiment 4 the average z-score for the three objects of 275 g was set to represent 275 g and the difference in z-score between the small and large solid objects divided by the physical mass difference was used to convert the z-scores into units of grams.

## Additional Information

**How to cite this article**: Plaisier, M. A. and Smeets, J. B.J. Object size can influence perceived weight independent of visual estimates of the volume of material. *Sci. Rep.* **5**, 17719; doi: 10.1038/srep17719 (2015).

## References

[b1] Ross H. E. When is weight not illusory? Q. J. Exp. Psychol. 21, 346–355 (1969). 5378275 10.1080/14640746908400230

[b2] Charpentier A. Analyse experimentale: De quelques elements de la sensation de poids. Arch. de Physiol. Norm. et Path., 3, 122–135 (1891).

[b3] Buckingham G. Getting a grip on heaviness perception: a review of weight illusions and their probable causes. Exp. Brain Res. 232, 1623–1629 (2014). 24691760 10.1007/s00221-014-3926-9

[b4] Buckingham G. & Goodale M. A. Lifting without Seeing: The Role of Vision in Perceiving and Acting upon the Size Weight Illusion. PLOS ONE, 5, e9709 (2010). 20300575 10.1371/journal.pone.0009709 PMC2837753

[b5] Plaisier M. A. & Smeets J. B. J. Mass Is All That Matters in the Size-Weight Illusion. PLOS ONE, 7, e42518 (2012). 22912704 10.1371/journal.pone.0042518 PMC3415412

[b6] Platkiewicz J. & Hayward V. Perception-action dissociation generalizes to the size-inertia illusion. J. Neurophysiol. 111, 1409–1416 (2014). 24401709 10.1152/jn.00557.2013 PMC3962618

[b7] Flanagan J. R., Bittner J. P. & Johansson R. S. Experience Can Change Distinct Size-Weight Priors Engaged in Lifting Objects and Judging their Weights. Curr. Biol. 8, 1742–1747 (2008). 19026545 10.1016/j.cub.2008.09.042

[b8] Zwislocki J. J. & Goodman D. A. Absolute scaling of sensory magnitudes -a validation. Percept. Psychophys. 28, 28–38 (1980). 7413407 10.3758/bf03204312

[b9] Ellis R. R. & Lederman S. J. The role of haptic versus visual volume cues in the size weight illusion. Percept. Psychophys. 53, 315–324 (1993). 8483695 10.3758/bf03205186

[b10] Masin S. & Crestoni L. Experimental Demonstration Of The Sensory Basis Of The Size-Weight Illusion. Percept. Psychophys. 44, 309–312 (1988). 3226877 10.3758/bf03210411

[b11] Flanagan J. R. & Bandomir C. A. Coming to grips with weight perception: effects of grasp configuration on perceived heaviness. Percept. Psychophys. 62, 1204–1219 (2000). 11019617 10.3758/bf03212123

[b12] Amazeen E. L. & Turvey M. T. Weight Perception and the Haptic Size-Weight Illusion Are Functions of the Inertia Tensor. J. Exp. Psychol. Human Perc. Perf. 22, 213–232 (1996). 10.1037//0096-1523.22.1.213 8742263

[b13] Buckingham G. & Goodale M. A., Size Matters: A Single Representation Underlies Our Perceptions of Heaviness in the Size-Weight Illusion. PLoS ONE 8, e54709 (2013). 23372759 10.1371/journal.pone.0054709 PMC3553013

[b14] Grandy M. S. & Westwood D. A., Opposite perceptual and sensorimotor responses to a size-weight illusion. J. Neurophysiol. 95, 3887–3892 (2006). 16641383 10.1152/jn.00851.2005

[b15] Chouinard P. A. *et al.* Dissociable neural mechanisms for determining the perceived heaviness of objects and the predicted weight of objects during lifting: an fMRI investigation of the size-weight illusion. Neuroimage 44, 200–12 (2009). 18801445 10.1016/j.neuroimage.2008.08.023

[b16] Peters M. A., Balzer J. & Shams L., Smaller = denser, and the brain knows it: natural statistics of object density shape weight expectations. PLoS ONE 10, e0119794 (2015). 25768977 10.1371/journal.pone.0119794 PMC4358826

[b17] Kahrimanovic M., Bergmann Tiest W. M. & Kappers A. M. L. Discrimination thresholds for haptic perception of volume, surface area, and weight. Atten. Percept. Psychophys. 73, 2649–56 (2011). 21870206 10.3758/s13414-011-0202-y PMC3222810

